# Rothmund-Thomson Syndrome: Insights from New Patients on the Genetic Variability Underpinning Clinical Presentation and Cancer Outcome

**DOI:** 10.3390/ijms19041103

**Published:** 2018-04-06

**Authors:** Elisa A. Colombo, Andrea Locatelli, Laura Cubells Sánchez, Sara Romeo, Nursel H. Elcioglu, Isabelle Maystadt, Altea Esteve Martínez, Alessandra Sironi, Laura Fontana, Palma Finelli, Cristina Gervasini, Vanna Pecile, Lidia Larizza

**Affiliations:** 1 Dipartimento di Scienze della Salute, Università degli Studi di Milano, 20142 Milan, Italy; laura.fontana@unimi.it (L.F.), cristina.gervasini@unimi.it (C.G.); 2 UO Dermatologia e Venereologia, Asst Papa Giovanni XXIII, 24127 Bergamo, Italy; anloc@tiscali.it; 3 Department of Dermatology, Consorcio Hospital General Universitario de Valencia, 46014 Valencia, Spain; laura_cubells@hotmail.com (L.C.S.), aemder@gmail.com (A.E.M.); 4 Institute of Clinical Sciences, Imperial College London, London W12 0NN, UK; s.romeo@lms.mrc.ac.uk; 5 MRC London Institute of Medical Sciences, Imperial College London, W12 0NN London, UK; 6 Department of Pediatric Genetics, Marmara University Medical School, 34890 Istanbul, Turkey; nelcioglu2@yahoo.com; 7 Department of Pediatrics, Eastern Mediterranean University, Mersin 10 Cyprus, Turkey; 8 Centre de Génétique Humaine, Institut de Pathologie et de Génétique, 6041 Charleroi (Gosselies), Belgium; isabelle.maystadt@ipg.be; 9 Laboratory of Medical Cytogenetics and Molecular Genetics, IRCCS Istituto Auxologico Italiano, 20149 Milan, Italy; alessandra.sironi@unimi.it (A.S.); palma.finelli@unimi.it (P.F.); l.larizza@auxologico.it (L.L.); 10 Department of Medical Biotechnology and Translational Medicine, University of Milan, 20133 Milan, Italy; 11 Institute for Maternal and Child Health, Foundation IRCCS Burlo Garofolo Institute, 34137 Trieste, Italy; vanna.pecile@burlo.trieste.it

**Keywords:** Rothmund-Thomson syndrome, *RECQL4*, clinical expressivity, transcript analysis, osteosarcoma outcome

## Abstract

Biallelic mutations in *RECQL4* gene, a caretaker of the genome, cause Rothmund-Thomson type-II syndrome (RTS-II) and confer increased cancer risk if they damage the helicase domain. We describe five families exemplifying clinical and allelic heterogeneity of RTS-II, and report the effect of pathogenic *RECQL4* variants by *in silico* predictions and transcripts analyses. Complete phenotype of patients #39 and #42 whose affected siblings developed osteosarcoma correlates with their c.[1048_1049del], c.[1878+32_1878+55del] and c.[1568G>C;1573delT], c.[3021_3022del] variants which damage the helicase domain. Literature survey highlights enrichment of these variants affecting the helicase domain in patients with cancer outcome raising the issue of strict oncological surveillance. Conversely, patients #29 and #19 have a mild phenotype and carry, respectively, the unreported homozygous c.3265G>T and c.3054A>G variants, both sparing the helicase domain. Finally, despite matching several criteria for RTS clinical diagnosis, patient #38 is heterozygous for c.2412_2414del; no pathogenic CNVs out of those evidenced by high-resolution CGH-array, emerged as contributors to her phenotype.

## 1. Introduction

Rothmund-Thomson syndrome (RTS, MIM#268400) is a rare autosomal recessive genodermatosis, characterized by wide clinical expressivity, primarily accounted by locus heterogeneity, but to be further deepened considering allele heterogeneity and genetic modifiers [1]. Approximately 400 RTS patients are reported in the literature and generally all present as a hallmark sign the cutaneous erythematosus rash appearing within the first two years of life at sun-exposed areas, mainly on the face, then evolving into post-inflammatory chronic poikiloderma, a permanent lesion characterized by skin atrophy with hypo- and hyper-pigmented areas and telangiectasia [2]. Other common features manifested in early childhood are growth delay, hyperkeratosis and sparse and thin hair, eyelashes and eyebrows, while premature aging is observed in adult age [2]. However, only two-thirds of clinically diagnosed patients have biallelic alterations in *RECQL4* gene (MIM#603780) which encodes a protein belonging to RecQ helicase family, represented in human by five members (RECQL1, BLM, WRN, RECQL4 and RECQL5) all with a fundamental role in safeguarding genome stability [3]. The predominant subset of *RECQL4*-mutated patients represents RTS type-II or Thomson-like entity, characterized by poikiloderma, skeletal defects and cancer predisposition (osteosarcoma (OS) and less frequently squamous cell carcinoma of the skin, hematological tumors and other malignancies) [4,5]. The RTS type-I or Rothmund-type includes by definition patients negative to *RECQL4* mutations who present like the RTS-II ones poikiloderma, ectodermal dysplasia and growth retardation but differ in displaying the hallmark sign of bilateral juvenile cataracts: the RTS-I genetic defect is so far unknown [1].

Intriguingly, the most fearful sign of cancer predisposition, together with genome instability and premature aging, is shared by RTS-II, Bloom (MIM#210900) and Werner (MIM#277700) syndromes, all caused by defective functioning of RecQ helicases [6]. In addition to RTS, *RECQL4* biallelic alterations are also responsible for Baller-Gerold (BGS, MIM#218600) and RAPADILINO (MIM#266280) syndromes. All three *RECQL4*-associated diseases are clinically characterized by growth delay, bone alterations and cancer predisposition, and the underlying biallelic pathogenic variants exert their effect by a loss-of-function mechanism [7]. RTS is the most prevalent and hence best characterized entity, while RAPADILINO is a Finnish inheritance disorder so far reported in about 20 cases [8] and BGS is even rarer with less than a dozen of cases confirmed by molecular diagnosis [9].

The *RECQL4* gene, mapping to 8q24, encodes for a 1208 amino acids DNA helicase, a multi-domain protein with a multi-functional role in essential processes of DNA metabolism, including DNA replication [10], DNA repair of double-strand breaks [11], nucleotide excision repair [12] and base excision repair [13], telomere maintenance [14], p53 transport to mitochondrion [15] and mitochondrial DNA biogenesis [16].

Like the other members of RecQ family, RECQL4 protein is characterized by the central highly conserved RecQ helicase domain (spanning amino acid residues 489–850 and encoded by exons 8–15), while the N- and the C-terminal are unique among the family members. The RECQL4 N-terminus, a Sld2-like domain (1–388 aa residues), essential for the initiation of DNA replication shows at least two nuclear targeting signals (encompassing amino acids 37–66 and 363–492) [3,17]; a stretch of lysine residues (aa 376–386) subject to p300 acetylation [18] and a mitochondrial localization sequence (first 84 amino acids) [15]. At the C-terminal a R4ZBD domain (aa 836–1045) [19], structurally different but functionally comparable to RQC domain [20], and potential nuclear export signals are found [21].

The involvement of different RECQL4 domains in carrying on the multiple functions needed for the safeguard of genome stability predicts a notable heterogeneity of mutant alleles depending on their intragenic location and their combination, considering the known prevalence of compound heterozygous versus homozygous genotypes reported for RTS patients [1]. While pathogenic variants disrupting the helicase domain are classified as deleterious for RECQL4 as well as for WRN and BLM, variants in the N-terminus region of RECQL4, which for its role in DNA replication initiation and replication fork progression is indispensable for cell viability [22], have likely a sub-lethal effect, consistent with the few mutations, never in the homozygous condition, identified in this region [7]. Current poor knowledge of the specific roles of RECQL4 domains and subdomains precludes to rank different mutations according to the compromised domains and functions, but the increasing number of novel characterized pathogenic variants makes it worthwhile to detail the spectrum of the observed RTS phenotypes, especially as regards cancer outcome.

We herein report on five unrelated families with RTS-II affected members who highlight the huge variability of the clinical presentation depending on the strength of the underlying pathogenic variants and their homozygous or heterozygous combination. We have characterized the causative *RECQL4* genetic lesions and have explored their pathogenic effect by in silico predictions and transcripts analyses to address mutation-phenotype correlation, especially in relation to cancer development.

## 2. Results

**Figure 1:**
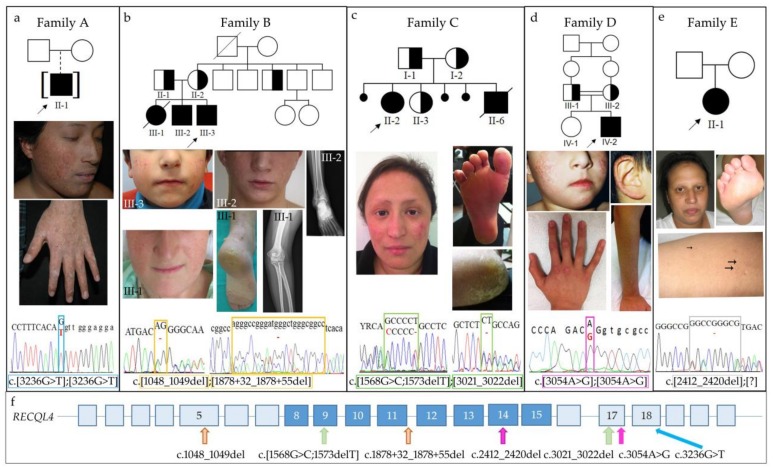
Clinical and molecular characterization of the five (A, B, C, D, E) RTS families. For each family, pedigree is furnished at the top, pictures of the major features in the middle and electropherograms of *RECQL4* pathogenic variants in the bottom panels. (**a**) Patient #29 (family A) at age 16y: poikiloderma on face and hand, sparse eyebrows, hyperkeratotic areas on the knuckles and onychodystrophy can be observed. The homozygous c.3236G>T transversion in exon 18 is squared in blue. (**b**) Facial poikiloderma of the living siblings of family B, III-3 (#39) and III-2 at 5 and 19 years, respectively; right ankle radiography of III-2 showing a lytic focal eccentric lesion (21 × 20 × 40 mm) with “bubble” pattern and geographical margins on the side of the distal epiphyseal-metaphyseal region of the tibia. Facial poikiloderma and severe plantar hyperkeratosis of the deceased elder sister III-1 at 23y and left elbow radiography of III-1 showing a lytic lesion in the olecranon with permeative appearance and ill-defined contours with neoplastic appearance of bone matrix. The sibship c.1048_1049del in exon 5 and c.1878+32_1878+55del in intron 11 variants are squared in orange. (**c**) Patient #42 (family C): poikiloderma, sparse eyebrows and absent eyelashes can be appreciated on the face; notable plantar hyperkeratosis, in particular on the heel, is observed. Exon 9 c.[1568G>C;1573delT] and exon 17 c.3021_3022del alterations are squared in green. (**d**) Patient #19 (family D): poikiloderma on cheeks and ear (age 5y); poikiloderma on forearm and keratoderma over the phalangeal joints can be seen (age 16y). The c.3054A>G pathogenic variant affecting the penultimate nucleotide of exon 17 is squared in purple. (**e**) Patient #38 (family E): face image showing fine and sparse hair, eyelashes and eyebrows. Plantar hyperkeratosis and cutaneous small white papule (arrowed) on the forearm can be observed. The c.2412_2420del alteration in exon 14 is evidenced by a grey square. (**f**) *RECQL4* intragenic location of the pathogenic variants is arrowed. A color-code is used to match mutations to index cases of the different families. RECQL4 helicase domain is shaded in blue.

Five families (A, B, C, D, E) with one or more siblings clinically diagnosed with RTS were referred to our laboratory for *RECQL4* molecular analysis. Genealogic, clinical and molecular data are provided in Figure 1, panels a, b, c, d and e, respectively.

Details on the cutaneous, skeletal, gastrointestinal findings, cancer development and *RECQL4* genotypes, either assessed or inferred, are provided for all patients in Table 1.

### 2.1. Clinical Report of RTS Patients

#### 2.1.1. Family A

Patient #29 (II-1) is a boy of Ecuadorian origin, who was adopted by an Italian couple. The adoptive parents referred to poikiloderma onset at 6–7 months, first on cheeks, then spreading to arms and forearms. They also noted photosensitivity and growth delay starting at pediatric age. At the first dermatologic evaluation at age 14 years (y), the boy showed poikiloderma on sun-exposed body sites (face, limbs and buttocks) and slight hypotrichosis of lateral third of eyebrows (Figure 1a).

He presented linear hyperkeratotic papule on the ventro-dorsal junction of both hands and feet, some hyperkeratotic areas on the palms, soles and on the knuckles and onychodystrophy (Figure 1a). He did not show radial ray defects, but osteosonography evidenced phalangeal hypodensity. At eye examination, keratoconus was observed (Table 1).

Stature at age of 16 years was 157 cm (<3rd percentile) and weight 60.6 kg (25th–50th percentile). Check-up for skin and bone cancer as well as hematological cancer is negative at the current age of 19 years.

#### 2.1.2. Family B

The Spanish family includes three affected siblings, a female (III-1) deceased at age 24 years and two males, 21 (III-2) and 6 (III-3) years old (Figure 1b).

According to the clinical records, the elder sister III-1 manifested poikiloderma, photosensitivity and delayed growth since the first year of life. The parents also reported constipation and gastro-esophageal reflux. At 23 years, she presented with facial malar erythema with surface telangiectasia, sparse eyelashes, thick eyebrows, hyperkeratotic lesions on both heels and on the skin over the Achilles tendon (Table 1). Deepened investigations for pain in her left elbow revealed a high-grade OS (T2 N0 MO G2), stage IIB that invaded fibroadipose and musculoskeletal soft tissues. A whole-body bone scintigraphy study ruled out metastases. Treatment with the chemotherapy regimen Adriamycin (25 mg/m^2^ for 3 days), Ifosfamide (1800 mg/m^2^ for 5 days) followed by 5-methotrexate (12 g/m^2^), Cisplatin-VP16 plus Mifamurtide in two doses was administered. Soon after chemotherapy, she had diarrhea, colic-type pain, elevated liver enzymes and developed fever, oral mucositis grade 2 and persistent bone marrow aplasia. Sepsis caused by *Pseudomonas* was diagnosed. Despite treatment with antibiotics, the patient health continued to deteriorate: she developed pneumonia, severe bone marrow aplasia, liver failure, severe mucositis with ulcers and extensive oral erythema that precluded food swallowing, and she presented large areas of denuded skin. Lyell’s syndrome was diagnosed, for which she received intravenous immunoglobulin treatment at a dose of 1 g/kg/day for 4 days. Unfortunately, her condition continued to worsen and she died a few weeks later. Test for methylenetetrahydrofolate reductase (*MTHFR*) revealed that she was homozygous for the c.1298A>C polymorphism (p.(Glu429Ala)), variant associated with toxicity and adverse drug events after methotrexate treatment [23].

Clinical history of the elder brother (III-2) recalls diffuse erythema on both cheeks at 6 months, which spread to the entire face and evolved into the typical poikiloderma with hypo- and hyper-pigmented skin areas and telangiectasia (Figure 1b). The patient manifested severe photosensitivity and growth delay. There was no history of gastrointestinal disease, no plantar hyperkeratotic lesions and sexual development was normal. On last physical examination at age 21 y, he was found to be of short stature, with fine hair and madarosis (Table 1). Following persistent pain at his ankle, a radiographic study was performed evidencing an eccentric, lytic focal lesion on the lateral side of the right tibia epiphyseal-metaphyseal region. In addition, generalized low bone density and tibio-talar joint degenerative changes were detected. Bone biopsy and immunohistochemical studies allowed the diagnosis of low-grade fibroblastic OS. The patient underwent radical oncological surgery, but no adjuvant chemotherapy was administered. One year after the amputation, nuclear magnetic resonance revealed a 29 mm × 29 mm solid tumor in the popliteal space of the right knee. Bone biopsy revealed a completely necrosed high-grade sarcoma and the supracondylar limb was amputated. The patient is currently waiting to commence adjuvant chemotherapy treatment. Like his sister, he was found homozygous for the c.1298A>C polymorphism (p.(Glu429Ala)) of *MTHFR* gene.

The youngest sibling III-3 (#39) is a 6-year-old male and presented with reticulated erythema limited to both cheeks (Figure 1b) since he was a year and a half old, but he did not develop photosensitivity. There is no history of growth delay, skeletal and gastrointestinal abnormalities; hair, eyelashes and eyebrows are normal (Table 1).

#### 2.1.3. Family C

The index case II-2 (#42) is a 31-year-old female from South Italy born to unrelated parents originated from two closed small villages. One abortion (II-4) and two late fetal deaths in utero (II-1 and II-5) are recorded (Figure 1c).

She has poikiloderma on the cheeks (Figure 1c), sparse eyebrows and absent eyelashes, nail dysplasia, plantar hyperkeratosis and dental defects (Table 1). Since infancy, she manifested photosensitivity, growth delay and suffered of diarrhea. Lacrimal duct obstruction was corrected. X-ray investigations performed at age 22 years showed several osteosclerosis nodules and cystic-like lesions on both femur heads, acetabula and on ischiopubic and iliopubic branches. At age 27 she had an accidental fracture of the fifth metatarsal bone. At age 31 during her first pregnancy, she developed gestational diabetes. Several threats of miscarriage occurred during pregnancy. The child was born at 33 weeks with an emergency cesarean section for fetal distress, and he presented with low weight and length (both 3rd centile) and congenital hydrocephalus, which has been successfully treated.

At the last examination, she has not developed any cancer, while her younger brother (II-6, Figure 1c) developed at age 14 an isolated OS at the right distal ulna and, 3 years later, another OS at the femur head, causing his demise at age 18 years. According to the clinical history, the RTS clinical phenotype of the brother was more severe than that of his sister (II-2) (Table 1). Indeed, he presented at birth with hydrocephalus and renal tubulopathy, then he developed diarrhea, food intolerance, iridocyclitis and chronic otitis. Besides early onset poikiloderma, absent/sparse hair, eyelashes, eyebrows, photosensitivity, dental defects, palmo-plantar hyperkeratosis, he presented a severe growth delay which was treated with growth hormone (GH) (Geneteopin) at age 6 years for 6 months with positive results.

#### 2.1.4. Family D

Patient #19 (IV-2) is the second child of Turkish consanguineous (first cousins) parents (Figure 1d). At his first examination at age 3.5 years, he displayed poikiloderma on sun-exposed areas, referred to be present since age 2, and keratoderma over phalangeal joints, palms, elbows and knees. Skin biopsy at age 3 revealed sub-epithelial melanin deposition, vacuolar changes at basal layers and rare keratinocyte necrosis raising the clinical diagnosis of RTS. He suffered from recurrent middle ears infections and immunological evaluation revealed IgA deficiency, treated with monthly IV-IG infusions for a while. At age 9, he suffered from a knee arthritis (Table 1). At age 16, corresponding to last examination, the patient presented poikiloderma on cheeks, ears, forearms and knees (Figure 1d), keratoderma on palms and joints and teeth with irregular ends. Poikilodermatous skin lesions were not spread or getting worse with age. Neither eye, hair or nail problems were present that time. He was using high factor sunblock for protecting his affected skin parts.

#### 2.1.5. Family E

Patient #38 is a Belgian woman that presented with small stature (adult height: 159 cm), sparse scalp hair, eyelashes and eyebrows, enamel defect and hyperkeratosis of the soles of the feet (Figure 1e). She has no typical poikiloderma but small white lesions of the skin and swelling of the lower limbs. She has hypogonadism, generalized osteopenia but she does not show nail abnormalities. She suffered of diarrhea in infancy that resolved spontaneously with age. In addition chronic anemia (Hb 9.5 g/dL), a moderate hepatosiderosis, hyperferritinemia with negative hemochromatosis genetic test, hypercholesterolemia and insulin resistance were reported (Table 1). At the age of 12 years, she developed an alveolar rhabdomyosarcoma grade III of the lower limb, successfully treated with chemotherapy and surgery.

### 2.2. Molecular Characterization of RTS Patients

Biallelic alterations in *RECQL4* gene were found in all patients from all families with the exception of patient #38 where only one *RECQL4* heterozygous alteration was detected.

**Figure 2:**
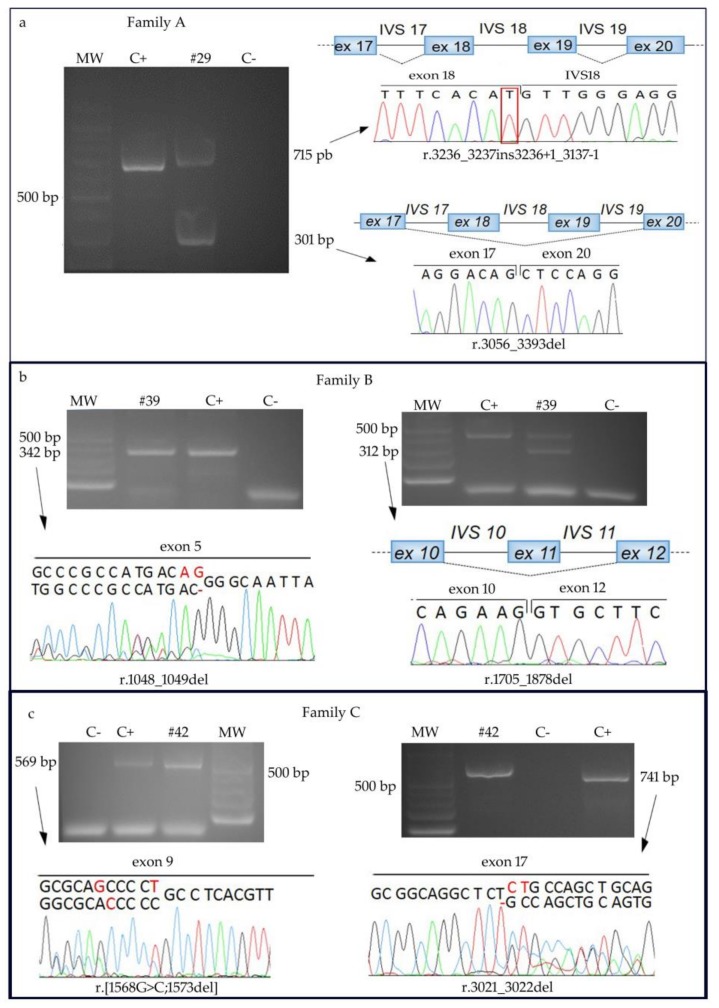
*RECQL4* transcript analyses in RTS families A, B, C. (**a**) Agarose gel showing two aberrant RT-PCR products in patient #29 homozygous for c.3236G>T alteration: sequencing of the slower migrating band shows intron 18 retention (76 nucleotides) while electropherogram obtained from the faster migrating band reveals skipping of exons 18 (181 nt) and 19 (157 nt). (**b**) Left panel: electopherogram of the RT-PCR product (exons 5–7) including the heterozygous c.1048_1049del of patient #39 highlights the lack of two nucleotides (in red). The wild-type sequence refers to the other allele of the patient carrying a downstream deletion. Right panel: agarose gel of the RT-PCR product of exons 9–13 amplicon and electropherogram of the faster migrating band showing a mis-spliced transcript lacking exon 11 (174 nt) due to the IVS11 c.1878+32_1878+55del deletion. The wild-type amplicon refers to the other patient allele carrying an upstream deletion. (**c**) Left panel: electropherogram of the RT-PCR product (exons 7–12) including the heterozygous c.[1568G>C;1573delT] of patient #42 highlights the out-of-frame change (in red) transversion and the in cis close deletion. The wild-type sequence refers to the other allele of the patient carrying the downstream exon 17 deletion. Right panel: electropherogram of the RT-PCR product (exons 15–20) including the heterozygous c.3021_3022del of the patient. The wild-type sequence refers to the other patient allele with the upstream exon 9 transversion/deletion. MW: molecular weight markers; C+: positive control (cDNA of a healthy individual); C-: negative control (no template added).

Patient #29 carries the homozygous c.3236G>T substitution affecting the last nucleotide of exon 18 (Figure 1a). The unavailability of DNA from the biological parents precluded segregation analysis of the observed alteration. This yet unreported missense variant, which should lead to the substitution of the 1079 Serine residue, evolutionary conserved across mammals, with Isoleucine (p.(Ser1079Ile)) is predicted to be damaging by SIFT, Polyphen2, Provean algorithms and to have a moderate functional impact by Mutation Assessor. In addition, ESEFinder, Human Splicing Finder, Splice View and NetGene2 predict that the c.3236G>T causes detriment of the wild-type donor splice site, inhibiting intron 18 correct splicing. We confirmed these predictions by sequencing the RT-PCR amplicons visible on the agarose gel (Figure 2a, left panel). Indeed the 715 bp fragment includes the entire IVS18 sequence, while the faster migrating product of 301bp results from skipping of exons 18 and 19 (Figure 2a, right panel). In both cases, the reading frame is disrupted and a downstream premature termination codon is exposed predicting the truncated protein p.Ser1079MetfsTer17 and p.Gly1019AlafsTer95, respectively.

As regards family B, the *RECQL4* molecular study showed that both brothers are compound heterozygous for the paternally inherited c.1048_1049del in exon 5 and the maternal c.1878+32_1878+55del in IVS11 (Figure 1b). Both pathogenic variants are inferred to be present in the deceased elder sister III-1. Both deletions have been previously reported [8,24,25,26,27], but their effect at the transcript level has not been explored.

Sequencing of the cDNA amplified region between exon 5 and exon 7, which includes the c.1048_1049del deletion, revealed beyond the expected sequence, an aberrant transcript lacking two exon 5 nucleotides (Figure 2b), which is potentially translatable in the p.Arg350GlyfsTer21 protein.

As to the c.1878+32_1878+55del deletion in the small intron 11 (81 nucleotides), amplification of the cDNA portion (exons 9–13) allowed to retrieve two different *RECQL4* amplicons: the expected 486 bp fragment and a faster migrating 312 bp product (Figure 2b). Sequencing of the latter showed a non canonical exon 10–12 junction resulting from exon 11 skipping (Figure 2b). According to ESEFinder, the c.1878+32_1878+55del includes the branch site of IVS11 precluding, as well the small intron size, correct splicing. The skipped transcript maintains the reading frame and, if translated, would encode for a protein lacking 58 aa within the helicase domain (p.Ile569_Lys626del).

The index case II-2 of family C is compound heterozygous for the novel *RECQL4* exon 17 c.3021_3022del, inherited from her mother, and the highly recurrent alteration [8,26,28,29] of exon 9 c.[1568G>C;1573delT] inherited from her father (Figure 1c). The same mutations are inferred to be carried by the deceased younger brother (II-6), while the healthy younger sister (II-3) has inherited the maternal deletion (Figure 1c).

RT-PCR and sequencing of the *RECQL4* cDNA portions, encompassing exons 7–12 and 15–20, evidenced the presence of two aberrant transcripts carrying either exon 9 or exon 17 alterations, beyond wild-type sequences (Figure 2c). Both aberrant transcripts present a premature stop codon and, if translated, should give origin to truncated proteins, p.[Ser523Thr;Cys525AlafsTer33] and p.Cys1008ProfsTer24, respectively.

The homozygous c.3054A>G sequence change has been identified in patient #19 (Figure 1d). This silent mutation involving the penultimate nucleotide of exon 17 is not present in variant databases, and, according to in silico predictions of NetGene2 and Splice View it should cause mis-splicing due to decreased strength of the physiological donor splice site of intron 17. The Human splicing finder tool also predicts interference with splicing, by exonic splicing enhancer (ESE) modification.

High resolution CGH-array and Genome-wide SNP-array did not highlight rare pathogenic CNVs contributing to the RTS phenotype.

Finally, PCR amplifications of whole *RECQL4* region of patient #38 has permitted to identify only one heterozygous alteration in exon 14, the c.2412_2420del deletion (Figure 1e). This pathogenic variant is not reported in literature but is included in databases as variant rs766312203 with uncertain significance: allele frequency in ExAc is 0.00004628 (=1/21607). The aberrant transcript should be translated in a protein (p.(Ala805_Arg807del)) missing 3 amino acids within the RECQL4 helicase domain.

High resolution CGH-array analysis did not evidence unreported genomic rearrangements/CNVs with a possible contributing role in the observed phenotype (data not shown).

## 3. Discussion

The RTS-II patients herein described well summarize the remarkable clinical breadth of Rothmund-Thomson syndrome including mild as well as severe phenotypes at high risk for tumor development, primarily dependent on the pathogenic variants at the causative *RECQL4* gene.

Patients #19 and #29 presenting with a phenotype mainly restricted to cutaneous alterations, are homozygous for the unreported pathogenic variants c.3054A>G, in exon 17, and c.3236G>T, in exon 18, hence sparing the helicase domain, encoded by *RECQL4* exons 8-15. Both alterations lead to mis-splicing which is predicted by several softwares in the case of patient #19 and is demonstrated for patient #29 (Figure 2a). In both cases mis-splicing impacts the RECQL4 C-terminal region (RQC) which comprises a zinc-binding domain and a winged helix domain involved in protein–protein interactions and regulation of the helicase activity, respectively [20]. Identification of pathogenic variants affecting the RQC region, especially in homozygous state, is helpful to dissect the function of RECQL4 domains less explored than the key helicase domain, but involved in its regulation. We can infer by our young adult patients #19 and #29 presenting with an overall mild phenotype, that their splicing variants, though much less deleterious compared to truncating mutations, are able to reproduce all the main cutaneous findings of RTS, likely due to defective function of this RQC terminal region. Moreover, we also speculate that a putative residual helicase activity of the proteins encoded by the observed aberrant transcripts might contribute to the mild phenotype of patients #19 and #29.

Instead, a severe phenotype characterizes the affected individuals of families B and C. Osteosarcoma developed in two siblings of family B, found to be compound heterozygous for c.1048_1049del and c.1878+32_1878+55del deletions in exon 5 and intron 11, and in the index case of family C, carrying the recurrent complex c.[1568G>C;1573delT] and the novel c.3021_3022del alterations in exons 9 and 17.

Out of these pathogenic variants, two (c.[1568G>C;1573delT]; c.1878+32_1878+55del) directly impact and one (c.1048_1049del) leads to the lack of the helicase domain confirming the known assumption that patients with at least one *RECQL4* truncating mutation disrupting the helicase domain have an increased risk to develop osteosarcoma at young age (mean age 11 y) [2]. Moreover, these three alterations have been already reported in literature in RTS patients with tumor outcome, as surveyed in Table 2.

Both pathogenic variants of affected members from family B modify the helicase coding sequence and should contribute to cancer development. The IVS11 c.1878+32_1878+55del deletion has been reported only once in a patient who developed OS in childhood, while out of the eight cases in the literature with c.1048_1049del deletion in exon 5 only one adult patient developed Hodgkin’s lymphoma at 35 years: the others may not have developed tumors due their young age (<13 y) at the analysis timepoint. It is worth noting that the c.1048_1049del allele is transcribed in an aberrant transcript with a premature stop codon escaping, at least partially, nonsense mediated decay (NMD), as we detected it by RT-PCR analysis, while the transcript of the c.1878+32_1878+55del allele leading to the internal in-frame deletion of exon 11 should not trigger NMD.

Moreover, the transcript carrying r.1048_1049del deletion might potentially be translated into a prematurely truncated protein that is predicted to lack the helicase domain and the lysine-rich region of nuclear targeting signal 2, which should cause cytoplasmic RECQL4 accumulation upon acetylation of the lysine residues (K376, K380, K382, K385, K386) by the p300 acetyltransferase [18].

In family C, where only one of two affected siblings developed OS at young age, the helicase domain is perturbed by the paternal exon 9 c.[1568G>C;1573delT] alteration, which is definitely the most recurrent *RECQL4* pathogenic variant [1]. It has been associated to RTS and so far reported in 21 patients, seven of whom developed OS. Furthermore, c.[1568G>C;1573delT] has been found in heterozygous condition in one RAPADILINO patient [8] and 4 BGS siblings [8,31], comprising two fetuses and one child deceased at 3 years of age. The maternal c.3021_3022del in exon 17 is unreported and falls in gene region coding for RECQL4 C-terminus.

Table S1 provides a general overview of the *RECQL4* pathogenic variants detected to our knowledge in 35 patients who developed cancer presenting with all the three *RECQL4*-associated diseases (28 RTS, 6 RAPADILINO and 1 BGS patients) [8,26,27,28,30,32,33,34]. Out of 35 *RECQL4*-mutated patients, 34 carried at least one pathogenic variant affecting the helicase domain, confirming the association between deleterious mutations in the *RECQL4* gene and cancer predisposition first assessed by Wang in 2003 [35].

Switching back to the RTS cancer families herein described, consistent with the location and predicted effect of the pathogenic variants the cancer predisposition appears more penetrant in family B where two siblings developed OS versus one in family C, as a second RTS affected member is cancer free at current age of 31 years. Nevertheless in both families OS, occurring at two different sites, was fatal at young age to the affected sibling. Apart cancer outcome, the phenotype of affected members is complete and severe in both families, also including rare RTS findings, although intrafamilial variability (for instance in gastrointestinal signs in family B siblings) is apparent and can be only ascribed to modifiers loci not shared by the siblings.

Cancer has also occurred in patient #38 who however developed rhabdomyosarcoma, an RTS-unusual tumor, and displays atypical skin RTS phenotype. Though she manifests the additional clinical features required to establish the RTS diagnosis as sparse/absent hair/eyelashes/eyebrows, hyperkeratosis, growth delay, diarrhea in infancy, bone, teeth and nail defects, she does not show the classic poikiloderma. Even if representing the hallmark cutaneous defect of RTS, poikiloderma is quite rare in the *RECQL4*-related disorders RAPADILINO [36] and Baller-Gerold [9] making atypical skin changes in suspected RTS patients worth to be considered.

Patient #38 is also unsolved by molecular diagnosis as she is heterozygous carrier of a very rare c.2412_2420del pathogenic variant and is here included for sake of completeness, in keeping with the literature where at least 11 patients carrying only one heterozygous mutation have been described [26,29,35,37]. Interestingly, the c.2412_2420del alteration of patient #38 falls in the region encoded by exon 14, which undergoes a physiological alternative splicing. An increased amount of the alternative in-frame transcript, encoding for a likely functional protein lacking 66 amino acids of the helicase domain, has been detected in two RTS siblings characterized by mild phenotype carrying the c.2272C>T mutation which is abolished by exon 14 alternative splicing [38]. Unfortunately, patient #38 RNA was not available to investigate an expression change of the physiological alternative transcript r.2266_2463del.

In conclusion, deep phenotyping of a small cohort of patients with the ultra-rare Rothmund-Thomson type-II syndrome emphasizes the remarkably different clinical presentation with demand in some instances of patient management by an integrated team of professionals, including the oncologist. Characterization at DNA and RNA level of patients’ pathogenic variants is a valuable tool to predict their effect on overall phenotype and disease evolution, allowing for providing patients with oncological surveillance and personalized care of medical complications. Last, expanding the repertoire of *RECQL4* gene variants affecting protein domains which a scarcely known role is instrumental to address functional studies [39] aimed at precise mapping of intertwined functions of the multifunctional RECQL4 protein.

## 4. Materials and Methods

### 4.1. Biological Material from Affected and Healthy Individuals from RTS Families

Affected individuals of five unrelated families (A, B, C, D, E) with a clinical diagnosis of suspected or probable RTS were referred to our laboratory by clinical geneticists and dermatologists. All patients and their family members were enrolled in this study after obtainment of appropriate informed consent to genetic analysis and photos collection.

### 4.2. DNA Isolation and RECQL4 Mutational Analysis

Genomic DNA from peripheral blood of the index cases and their family members was isolated with Wizard Genomic DNA Purification Kit (Promega, Madison, WI, USA) according to standard protocols.

The entire *RECQL4* gene (NG_016430.1) (21 exons and 20 introns with the exception of IVS12 minisatellite) was amplified using GoTaq^®^ Flexi DNA polymerase (Promega) and primers listed in Table S2.

PCR products were sequenced according to the manufacture’s protocol using Big Dye Terminator v.3.1 Cycle Sequencing Kit (Applied Biosystems, Foster City, CA, USA) and primers listed in Table S2 on the ABI PRISM 3130 sequencer (Applied Biosystems). Electropherograms were analysed with ChromasPro software 1.7.4 (Technelysium Pty Ltd., South Brisbane, Australia) using the wild type sequence of the *RECQL4* gene (GenBank: NG_016430.1) as reference.

Sequence variants were described according to HGVS nomenclature guidelines (http://varnomen.hgvs.org/) [40].

Pathogenic variants were included in LOVD database (http://lovd.nl/RECQL4) [41].

### 4.3. Cell Cultures

EBV-transformed lymphoblastoid cell lines (LCLs) were established from peripheral blood lymphocytes of patient #42, her parents and five healthy controls.

LCLs were cultured in complete RPMI-1640 medium (EuroClone, Milano, Italy) supplemented with 10% fetal bovine serum (Lonza, Walkersville, MD, USA) and 1% penicillin, streptomycin and ampicillin in a 37 °C humidified incubator with 5% CO_2_.

### 4.4. RNA Isolation, RT-PCR and cDNA Analysis

TRI Reagent (Sigma, St Louis, MO, USA) was used to isolate total RNA of patients #29, #39, his affected brother and his parents, #42, healthy controls from white blood cells, and from LCLs of family C, according to manufacturer’s protocols.

After DNase I (RNase-free, New England Bio-Labs, Inc., Ipswich, MA, USA) treatment, 500 ng of total RNA were used to synthesize cDNA using the High Capacity cDNA Reverse Transcription Kit (Applied Biosystems) with random hexamers. All samples were reverse transcribed in two independent experiments.

Different fragments of *RECQL4* transcript were amplified using GoTaq® Flexi DNA polymerase (Promega) and primers listed in Table S3.

Amplicons were run on 2% agarose gel, the bands were eluted with MinElute Gel Extraction Kit (Qiagen, Milano, Italy) and then sequenced as described above.

Nucleotide sequences were compared to the major *RECQL4* transcript reference sequence [GenBank: NM_004260.3].

### 4.5. Bioinformatic Analyses

ExAC Browser of Broad Institute (http://exac.broadinstitute.org/gene/) and 1000 Genomes database (http://www.1000genomes.org/home) [42] were checked to assess the presence/absence of detected alterations in variations repositories.

To predict the effect of the identified mutations at protein level, the bioinformatics tools SIFT (http://sift.jcvi.org/) [43], Polyphen2 (http://genetics.bwh.harvard.edu/pph2/) [44], Provean (http://provean.jcvi.org/index.php) [45], Mutation Assessor (http://mutationassessor.org/r3/) [46] and Mutalyzer (https://mutalyzer.nl/) [47] were applied.

To predict the impact of mutation on splicing ESEFinder (http://rulai.cshl.edu/cgi-bin/tools/ESE3/esefinder.cgi?process=home) [48], Human Splicing Finder (http://www.fruitfly.org/seq_tools/splice.html) [49], Splice View (http://www.umd.be/HSF/), and NetGene2 (http://www.cbs.dtu.dk/services/NetGene2/) [50] were consulted.

### 4.6. High-Resolution CGH-Array

As regards patients #19 and #38, high-resolution array-based comparative genomic hybridization (CGH-array) analysis was performed, on 700 ng genomic blood DNA, using the high resolution SurePrint G3 Human CGH Microarray Kit 2 × 400 K in accordance with the manufacturer’s instructions. (Agilent Technologies, Palo Alto, CA, USA). Images were captured using the Agilent Feature Extraction 3.0.5.1 software and chromosomal profile was acquired using the ADM-2 algorithm provided by Agilent CytoGenomics v.3.0.6.6 software (Agilent Technologies).

Coordinates of Copy Number Variants (CNVs) referred to the Human Genome assembly GRCh37/hg19 (https://genome.ucsc.edu/). CNV classification was performed according to the Database of Genomic Variants (http://projects.tcag.ca/variation/, release March 2016) to exclude common polymorphic CNVs with a frequency >1% in healthy control subjects. The CNV classification by clinical relevance was performed according to the guidelines suggested by Miller [51] and successively by the American College of Medical Genetics [52].

### 4.7. SNP-Array

SNP-array analysis was performed on samples of family #19 using the Human OmniExpress Exome-8 Bead Chip (Illumina Inc., San Diego, CA, USA) containing 960,919 loci derived from phases I, II and III of the International HapMap project. The array includes over 274,000 functional exonic markers, delivering unparalleled coverage of putative functional exonic variant selected from 12,000 individual exome and whole-genome sequences.

A total of 200 ng of gDNA (50 ng/µL) for each sample was processed according to Illumina’s Infinium HD Assay Super protocol. Normalization of raw image intensity data, genotype clustering and individual sample genotype calls were performed using Illumina’s Genome Studio software v2011.1 (cnvpartition 3.2.0). The CNVs were mapped to the human reference genome hg19 and annotated with UCSC RefGene. Allele detection and genotype calling were performed with Genome Studio software, B allele frequencies (BAFs) and log R ratios were exported as text files for PennCNV analysis.

## Figures and Tables

**Table 1:** Clinical characteristics of affected individuals from RTS families.

Family	A	B			C		D	E
Pedigree position	II-1	III-1	III-2	III-3	II-2	II-6	IV-2	II-1
Index case code	#29	-	-	#39	#42	-	#19	#38
Birth/death	1995	1989-2013	1993	2008	1986	1994-2012	1997	1980
Sex	M	F	M	M	F	M	M	F
Origin	Ecuador	Spain			Italy		Turkey	Belgium
Growth delay	+	+	+	-	+	+	-	+
Poikiloderma	+	+	+	+	+	+	+	- *
Onset (age)	6–7 m	1 y	<1 y	18 m	At birth	At birth	2 y	-
First localization	Sun-exposed areas	Cheeks	Cheeks	Cheeks	Face	Cheeks	Sun-exposed areas	-
Hyperkeratosis	Palmo-plantar	Plantar	-	-	Plantar	Palmo-plantar	Palms and joints	Plantar
Photosensitivity	Only in infancy	+	+	-	+	+	-	-
Hair	Normal	Thin	Thin	Normal	Sparse	Sparse	Normal	Sparse
Eyelashes	Normal	Sparse	Sparse	Normal	Absent	Absent	Normal	Sparse
Eyebrows	Sparse	Normal	Absent	Normal	Sparse	Sparse	Normal	Sparse
Onychodystrophy	+	-	-	-	+	+	Only in infancy	-
Dental defects	-	-	-	-	+	+	Irregular end	Enamel defect
Skeletal anomalies	Low bone density	n.a.	Low bone density	-	Osteosclerosis; Cystic-like lesion	-	n.a.	Osteopenia
Gastrointestinal	-	Constipation GER	-	-	Diarrhea; Food intolerance in infancy	Diarrhea; Food intolerance in infancy	-	Diarrhea in infancy
Cancer (onset age)	-	Olecranon OS (23 y)	Ankle OS (19 y)	-	-	Ulnar OS (14 y); Femur OS (17 y)	-	Alveolar rhabdomyosarcoma (12 y)
Others	CD4/CD8 = 2.5 keratoconus	-	Tibiotalar joint degenerative changes	-	-	-	Recurrent middle ear infections; IgA deficiency; Knee arthritis	Hypogonadism; Chronic anemia; Hyper-ferritinemia; Hyper-cholesterolemia; Insulin resistance
*RECQL4* genotype	c.[3236G>T]; [3236G>T]	c.[1048_1049del]; [1878+32_1878+55del]			c.[1568G>C;1573delT]; [3021_3022del]		c.[3054A>G]; [3054A>G]	c.[2412_2420del]; [?]

* White nodular lesions on the skin and swelling; GER= Gastroesophageal reflux disease; OS: osteosarcoma; +: sign present; -: sign absent; n.a.: data not available.

**Table 2:** Survey of tumor incidence in literature RTS patients carrying the same pathogenic variants of our patients with osteosarcoma outcome (families B and C).

Patient Code	Age at Analysis	Cancer	Pathogenic Variant I		Pathogenic Variant II		Reference
FCP-195	1 y	-	c.1048_1049del	ex 5	p.(Q757Ter)	ex 14	[8]
Pt 10	13 y	-	c.1048_1049del	ex 5	p.(Q757Ter)	ex 14	[8]
Pt 13	4 y	-	c.1048_1049del	ex 5	p.(Gln800Ter)	ex 14	[8]
RTS	13 y	-	c.1048_1049del	ex 5	p.(Gln757Ter)	ex 14	[25]
RTS	6 y	-	c.1048_1049del	ex 5	c.1391-1G>A	IVS7	[24]
Pt 13	5 y	-	c.1048_1049del	ex 5	p.(Gln800Ter)	ex 14	[26]
RTS 1	34 y	HL 35 y	c.1048_1049del	ex 5	c.1391-1G>A	IVS7	[27]
RTS 2	5 y	-					
#39 III-1	24 y ^†^	OS 23 y	c.1048_1049del	ex 5	c.1878+32_1878+55del	IVS11	This work
#39 III-2	22 y	OS 19 y					
#39 III-3	7 y	-					
FCP-210	-	OS 8 y	c.1718delA	ex 11	c.1878+32_1878+55del	IVS11	[8]
#42 II-2	31 y	-	c.[1568G>C;1573delT]	ex 9	c.3021_3022del	ex 17	This work
#42 II-6	18 y ^†^	OS 14 y, 17 y					
AS517	-	OS 13 y	c.[1568G>C;1573delT]	ex 9	p.(Leu926Arg)	ex 16	[28]
AS518	10 y	-					
AS287	32 y	-	c.[1568G>C;1573delT]	ex 9	p.(Gln757Ter)	ex 14	[28]
RTS	14 y	OS 10 y	c.[1568G>C;1573delT]	ex 9	p.(Gln757Ter)	ex 14	[30]
II-1	21 y	OS 21 y	c.[1568G>C;1573delT]	ex 9	c.1391-1G>A	IVS7	[8]
II-2	9 y ^†^	OS 7 y					
FCP-129	-	OS 4 y	c.[1568G>C;1573delT]	ex 9	p.(Gln757Ter)	ex 14	[8]
FCP-153	-	OS 20 y	c.[1568G>C;1573delT]	ex 9	c.1391-1G>A	IVS7	[8]
FCP-153 sibling	-	OS 9 y					
Pt 1	19 y	-	c.[1568G>C;1573delT]	ex 9	c.2059-1G>C	IVS12	[8]
FCP-157	10 y	-	c.[1568G>C;1573delT]	ex 9	p.(Gln757Ter)	ex 14	[8]
FCP-167	14 y	-	c.[1568G>C;1573delT]	ex 9	c.3270delG	ex 19	[8]
FCP-175	2 y	-	c.[1568G>C;1573delT]	ex 9	p.(Gln1175Ter)	ex 21	[8]
Pt 1	9 y	-	c.[1568G>C;1573delT]	ex 9	p.(Arg1021Trp)	ex 18	[8]
Pt 9	12 y	-	c.[1568G>C;1573delT]	ex 9	c.84+6del16	IVS1	[8]
Pt 11	11 y	-	c.[1568G>C;1573delT]	ex 9	p.(Gln821Ter)	ex 14	[8]
Pt 2	6 y	-	c.[1568G>C;1573delT]	ex 9	p.(Cys511Arg)	ex 9	[29]
Pt 3	6 y	-	c.[1568G>C;1573delT]	ex 9	p.(Trp412Ter)	ex 6	[29]
Pt 6	21 y	-	c.[1568G>C;1573delT]	ex 9	c.2059-1G>A	IVS12	[29]
Pt 9	3 y	-	c.[1568G>C;1573delT]	ex 9	p.(Arg1021Trp)	ex 18	[29]
Pt 14	8 y	-	c.[1568G>C;1573delT]	ex 9	c.1930_1935dup	ex 12	[26]

†: demise; OS: osteosarcoma; HL: Hodgkin’s lymphoma.

## Supplementary Materials

The following are available online at http://www.mdpi.com/1422-0067/19/4/1103/s1.
