# Supplementary material for: Rothmund-Thomson Syndrome: Insights from New Patients on the Genetic Variability Underpinning Clinical Presentation and Cancer Outcome
Source: Int J Mol Sci. 2018 Apr 6;19(4):1103. doi: 10.3390/ijms19041103 (PMC5979380; doi:10.3390/ijms19041103)
Supplement: Supplementary file 1 [file ijms-19-01103-s001.docx]

**Rothmund-Thomson syndrome: insights from new patients on the genetic variability underpinning clinical presentation and cancer outcome**

**Elisa A. Colombo ^1,*^, Andrea Locatelli ^2^, Laura Cubells Sánchez ^3^, Sara Romeo ^4,5^, Nursel H Elcioglu ^6,7^, Isabelle Maystadt ^8^, Altea Esteve Martínez ^3^, Alessandra Sironi ^9,10^, Laura Fontana ^1^, Palma Finelli ^9,10^, Cristina Gervasini ^1^, Vanna Pecile ^11^ and Lidia Larizza ^9^.**

^1^ Dipartimento di Scienze della Salute, Università degli Studi di Milano, 20142 Milan, Italy;

^2^ UO Dermatologia e Venereologia, Asst Papa Giovanni XXIII, 24127 Bergamo, Italy;

^3^ Department of Dermatology, Consorcio Hospital General Universitario de Valencia, 46014 Valencia, Spain;

^4^ Institute of Clinical Sciences, Imperial College London, London W12 0NN, UK;

^5^ MRC London Institute of Medical Sciences, Imperial College London, London W12 0NN, UK;

^6^ Department of Pediatric Genetics, Marmara University Medical School, 34890 Istanbul, Turkey;

^7^ Department of Pediatrics, Eastern Mediterranean University, Cyprus, Mersin 10, Turkey;

^8^ Centre de Génétique Humaine, Institut de Pathologie et de Génétique, 6041 Charleroi (Gosselies), Belgium;

^9^ Laboratory of Medical Cytogenetics and Molecular Genetics, IRCCS Istituto Auxologico Italiano, 20149 Milan, Italy;

^10^ Department of Medical Biotechnology and Translational Medicine, University of Milan, 20133 Milan, Italy;

^11^ Institute for Maternal and Child Health, Foundation IRCCS Burlo Garofolo Institute, 34137 Trieste, Italy;

***** Correspondence: elisaadele.colombo@unimi.it; Tel.: +39-02-50323200

**Table S1.** *RECQL4* pathogenic variants in *RECQL4*-mutated patients who developed cancer

| **Tumors**  **(Age at onset)** | **Patient code** | **Pathogenic Variant I** | | **Pathogenic Variant II** | | **Ref.** |
| --- | --- | --- | --- | --- | --- | --- |
| *Patients with malignancy carrying at least one RECQL4 alteration affecting the helicase domain (exons 8-15)* | | | | | | |
| Lymphoma (24y) | RAPA r704 | c.806G>A | ex. 5 | c.1390+2del | IVS7 | [8] |
| HL (35y) | RTS 1 | c.1048_1049del | ex. 5 | c.1391-1G>A | IVS7 | [27] |
| OS (23y)  OS (19y) | III-1  III-2 | c.1048_1049del | ex. 5 | c.1878+32_1878+55del | IVS11 | **This work** |
| SCC and BSC (≤39y) | RTS Pt 15 | c.1078C>T | ex. 5 | c.1222C>T | ex. 6 | [26] |
| OS (15y) | RAPA r504 | c.1390+2del | IVS7 | c.1390+2del | IVS7 | [8] |
| OS (10y) | RAPA Pt 7 | c.1390+2del | IVS7 | c.1390+2del | IVS7 | [8] |
| Lymphoma (21y) Lymphoma (25y) | RAPA r903  RAPA r904 | c.1390+2del | IVS7 | c.1390+2del | IVS7 | [8] |
| Lymphoma (33y) | RAPA Pt 6 | c.1390+2del | IVS7 | c.3599_3600del | ex. 21 | [8] |
| OS (21y)  OS (7y) | RTS II-1  RTS II-2 | c.1391-1G>A | IVS7 | c.[1568G>C;1573delT]^a^ | ex. 9 | [8] |
| OS (20y)  OS (9y) | RTS FCP-153  and sibling | c.1391-1G>A | IVS7 | c.[1568G>C;1573delT]^a^ | ex. 9 | [8] |
| OS (≤2y) | RTS Pt 12 | c.1391-1G>A | IVS7 | c.2085del | ex. 13 | [26] |
| OS (11y)  OS (12y) | RTS FCP-102  and sibling | c.1483+25del | IVS8 | c.1483+25del | IVS8 | [8] |
| OS (14y)  MFH (15y) | RTS IV-4  RTS IV-5 | c.1483+27del | IVS8 | c.1483+27del | IVS8 | [8] |
| OS (10y) | RTS | c.[1568G>C;1573delT] | ex. 9 | c.2269C>T | ex. 14 | [30] |
| OS (4y) | RTS FCP-129 | c.[1568G>C;1573delT]^a^ | ex. 9 | c.2269C>T | ex. 14 | [8] |
| OS (12y) | RTS AS517 | c.[1568G>C;1573delT]^a^ | ex. 9 | c.2269C>T | ex. 14 | [8,28] |
| OS (14y; 17y) | Family C II-6 | c.[1568G>C;1573delT] | ex. 9 | c.3021_3022del | ex. 17 | **This work** |
| OS (31y)  OS (15y) | RTS II-3  RTS II-6 | c.1650del | ex. 10 | c.2269C>T | ex. 14 | [8] |
| Lymphoma (9y)  OS (14y)  Leukemia (21y) | RTS | c.1704+1G>A | IVS10 | c.1919_1924del | ex. 12 | [32] |
| OS (8y) | RTS FCP-210 | c.1718delA | ex. 11 | c.1878+32del | VS11 | [8] |
| OS (7y) | RTS FCP-136 | c.1878+5G>A | IVS11 | c.2476C>T | ex. 15 | [8] |
| Lymphoma (2y) | RTS Pt 8 | c.1913T>C | ex. 12 | c.2419ins5 | ex. 14 | [8] |
| OS (17y) | RTS | c.2232_3007delC^b^ | ex. 14-17 | c.2232_3007del^b^ | ex. 14-17 | [33] |
| OS (9y) | RTS FCP-125 | c.2269C>T | ex. 14 | c.2269C>T | ex. 14 | [8] |
| Lymphoma (2.5y) | BGS | c.2492_2493del | ex. 15 | c.2506_2518del | ex. 15 | [34] |
| OS (19y) | RST FCP-191 | c.2492_2493del | ex. 15 | - | - | [8] |
| OS (13y) | RTS FCP-114 | c.2547_2548del | ex. 15 | -^c^ | - | [8] |
| *Patients with malignancy carrying RECQL4 alterations downstream the helicase domain (exons 8-15)* | | | | | | |
| OS (3y) | RTS FCP-203 | c.3072_3073del | ex. 18 | c.3276del | ex. 19 | [8] |

a: alteration reported as c.1573del in the original paper; b:alteration reported as g.4428_5437del (exons 14-18) in the original paper; c: three different amino acid substitutions (p.Arg522Cys, p.Val799Met and p.Pro1170Leu), none proven to be pathogenic.

HL: Hodgkin’s lymphoma; OS: osteosarcoma; SCC: squamous cell carcinoma; BSC: basal cell carcinoma; MFH: malignant fibrous histiocytoma.

RTS: Rothmund-Thomson syndrome; RAPA: RAPADILINO syndrome; BGS: Baller-Gerold syndrome.

**Table S2.** Primers used for amplification and sequencing of *RECQL4* gene

| ***RECQL4* AMPLICONS** | **PRIMER (5’ TO 3’)** | **LENGTH (bp)** |
| --- | --- | --- |
| 5’ UTR - IVS3 | **F:** TTGACGCCTCCCATTGGCT  **R:** TTGGTCGCAGCCCGATTCA | 815 |
| IVS3 - Exon 5 | **F:** AGAACTTGGGAGGGGGACTG  **R:** CACTGTGACATCGCTGTAACC | 791 |
| Exon 5 - IVS5 | **F:** GCAGAAAAAGTCAGTGATGAGC  **R:** TGGGCGGGAAATACGGGAGG | 747 |
| IVS5 - Exon 7 | **F:** CATTCCCTTTCCCTCCCCTCA  **R:** CTGCTCACCTGCCAACTGCCC | 644 |
| IVS6 - IVS9 | **F:** CTCCCATTCTACCCTCTCCT  **R:** CTGCCTTTGACCTGCTGCCA | 709 |
| Exon 9 - Exon 10 | **F:** TCTCTCCCCTGCTGCTGTCACTC  **R:** GATTCCCGTTGCTTCCTGGT | 513 |
| IVS9 - IVS11 | **F:** GGGCTGGGCTGGCGTATG  **R:** CCGCCCACCCCAGTTCACAT | 409 |
| Exon 11 - IVS12 | **F:** CAGTTGCTTTTGCCTGCATT  **R:** ACCTGGTCTGTGTCCCTGTC | 396 |
| IVS12 - Exon 15 | **F:** CTCCTCATCAGGCACTGTTG  **Fseq:** ATGAAGGCTCGCTGTACCC  **R:** GAGGACACAGAGCGGATCG  **Rseq:** CCTCTTCACAGCCAGGAAGT | 850 |
| IVS13 - IVS16 | **F:** CCCATCCCACTGACCATCT  **Fseq:** AGCCCCCAGTGGTCCACC  **R:** CTCCAACCTCGTCTCCAACT  **Rseq:** AGGAAGAGGTGGCAGTGGG | 912 |
| Exon 16 - IVS19 | **F:** GCGACCACCTATACCCATTG  **R:** CATCCACAGAGCAAGCCCC | 873 |
| IVS18 - Exon 21 | **F:** TCCTCCCCACAGCGTAGCC  **R:** ACTGCCCTAGCCTCTGACAA | 709 |
| IVS20 - 3’ UTR | **F:** AGGACCGACGCTTCTGGAG  **R:** TGTGCCTGGAATATGTGATGTG | 303 |

**Table S3. Primers used for amplification and sequencing of *RECQL4* transcripts**

| **FAMILY** | ***RECQL4* cDNA AMPLIFIED REGION** | **PRIMER**  **(5’ to 3’)** | **LENGTH**  **(bp)** |
| --- | --- | --- | --- |
| A | exon 16-17 junction - exon 20-21 junction | **F:** CCCACAGGTGTCCCCCTTT  **R:** GGTAGCAGGGGCTTCCGATG | 639 |
| B | exon 5 – exon 7 | **F:** AGCCCCTCCAGTCAAGCTAG  **R:** TGAAGGAACCAGTGGCTCAG | 342 |
|  | exon 9 –  exon 12-13 junction | **F:** TCTCTCCCCTGCTGTCACTC  **R:** AGCGTCAACAGTGCCTGGTC | 486 |
| C | exon 7 – exon 12 | **F:** ACCGTGCTGCCACTCTACTC  **R:** GAAGCAGTGCACGCCCAT | 569 |
|  | exon 15 –exon 20 | **F:** ACCGTACAGGCTTTGGACAT  **R:** GAACTTCTCCTCTGGCCTCA | 741 |

**References**

8. Siitonen, H. A.; Sotkasiira, J.; Biervliet, M.; Benmansour, A.; Capri, Y.; Cormier-Daire, V.; Crandall, B.; Hannula-Jouppi, K.; Hennekam, R.; Herzog, D.; Keymolen, K.; Lipsanen-Nyman, M.; Miny, P.; Plon, S. E.; Riedl, S.; Sarkar, A.; Vargas, F. R.; Verloes, A.; Wang, L. L.; Kääriäinen, H.; Kestilä, M. The mutation spectrum in RECQL4 diseases. *Eur. J. Hum. Genet. EJHG* **2009**, *17*, 151–158, doi:10.1038/ejhg.2008.154.

26. Suter, A.-A.; Itin, P.; Heinimann, K.; Ahmed, M.; Ashraf, T.; Fryssira, H.; Kini, U.; Lapunzina, P.; Miny, P.; Sommerlund, M.; Suri, M.; Vaeth, S.; Vasudevan, P.; Gallati, S. Rothmund-Thomson Syndrome: novel pathogenic mutations and frequencies of variants in the RECQL4 and USB1 (C16orf57) gene. *Mol. Genet. Genomic Med.* **2016**, *4*, 359–366, doi:10.1002/mgg3.209.

27. van Rij, M. C.; Grijsen, M. L.; Appelman-Dijkstra, N. M.; Hansson, K. B. M.; Ruivenkamp, C. a. L.; Mulder, K.; van Doorn, R.; Oranje, A. P.; Kant, S. G. Rothmund-Thomson syndrome and osteoma cutis in a patient previously diagnosed as COPS syndrome. *Eur. J. Pediatr.* **2017**, *176*, 279–283, doi:10.1007/s00431-016-2834-3.

28. Cabral, R. E. C.; Queille, S.; Bodemer, C.; de Prost, Y.; Neto, J. B. C.; Sarasin, A.; Daya-Grosjean, L. Identification of new RECQL4 mutations in Caucasian Rothmund-Thomson patients and analysis of sensitivity to a wide range of genotoxic agents. *Mutat. Res.* **2008**, *643*, 41–47, doi:10.1016/j.mrfmmm.2008.06.002.

30. Salih, A.; Inoue, S.; Onwuzurike, N. Rothmund-Thomson syndrome (RTS) with osteosarcoma due toRECQL4mutation. *BMJ Case Rep.* **2018**, *2018*, doi:10.1136/bcr-2017-222384.

32. Simon, T.; Kohlhase, J.; Wilhelm, C.; Kochanek, M.; De Carolis, B.; Berthold, F. Multiple malignant diseases in a patient with Rothmund-Thomson syndrome with RECQL4 mutations: Case report and literature review. *Am. J. Med. Genet. A.* **2010**, *152A*, 1575–1579, doi:10.1002/ajmg.a.33427.

33. Padhy, D.; Madhuri, V.; Pulimood, S. A.; Danda, S.; Walter, N. M.; Wang, L. L. Metatarsal osteosarcoma in Rothmund-Thomson syndrome: a case report. *J. Bone Joint Surg. Am.* **2010**, *92*, 726–730, doi:10.2106/JBJS.I.00478.

34. Debeljak, M.; Zver, A.; Jazbec, J. A patient with Baller-Gerold syndrome and midline NK/T lymphoma. *Am. J. Med. Genet. A.* **2009**, *149A*, 755–759, doi:10.1002/ajmg.a.32736.
